# The initial attempt at home hemodialysis in mainland China

**DOI:** 10.1186/s12882-022-03018-9

**Published:** 2022-12-06

**Authors:** Zhaohui Ni, Yijun Zhou, Renhua Lu, Jianxiao Shen, Leyi Gu, Shan Mou, Li Zhao, Haifen Zhang, Bin Zhang, Yan Fang, Wei Fang, Qin Wang, Weiming Zhang, Jidong Zhang, Weiping Li

**Affiliations:** 1 Department of Nephrology, Ren Ji Hospital, School of Medicine, Shanghai Jiaotong University, 160 Pujian Road, Shanghai, 200127 China; 2 Administration Department, Ren Ji Hospital, School of Medicine, Shanghai Jiaotong University, 160 Pujian Road, Shanghai, 200127 China

**Keywords:** Home hemodialysis, Trouble shooting, Mainland China

## Abstract

**Background:**

Observational studies have shown home hemodialysis (HHD) to be associated with better survival than facility hemodialysis (HD) and peritoneal dialysis (PD). Patients on HHD have reported higher quality of life and independence. HHD is considered to be an economical way to manage end-stage kidney disease (ESKD). The coronavirus disease 2019 pandemic has had a significant impact on patients with ESKD. Patients on HHD may have an advantage over in-center HD patients because of a lower risk of exposure to infection.

**Participants and methods:**

We enrolled HD patients from our dialysis center. We first established the HHD training center. The training center was approved by the Chinese government. Doctors, nurses and engineers train and assess patients separately. There are three forms of patient monitoring: home visits, internet remote monitoring, and outpatient services. Demographic and medical data included age, sex, blood pressure, and dialysis-related data. Laboratory tests were conducted in our central testing laboratory, including hemoglobin (Hgb), serum creatinine (Cr), urea nitrogen (BUN), uric acid (UA), albumin (Alb), calcium (Ca), phosphorus (P), parathyroid hormone (PTH), and brain natriuretic peptide (BNP) levels.

**Results:**

Six patients who underwent regular dialysis in the HD center of our hospital were selected for HHD training. We enrolled 6 patients, including 4 males and 2 females. The mean age of the patients was 47.5 (34.7-55.7) years, and the mean dialysis age was 33.5 (11.2-41.5) months. After an average of 16.0 (11.2-25.5) months of training, Alb, P and BNP levels were improved compared with the baseline values. After training, three patients returned home to begin independent HD. During the follow-up, there were no serious adverse events leading to hospitalization or death, but there were several adverse events. They were solved quickly by extra home visits of the technicians or online by remote monitoring. During the follow-up time, the laboratory indicators of all the patients, including Hgb, Alb, Ca, P, PTH, BNP, and β2-MG levels, remained stable before and after HHD treatment.

**Conclusion:**

HHD is feasible and safe for ESKD in China, but larger-scale and longer-term studies are needed for further confirmation.

## Introduction

Home hemodialysis (HHD) has been associated with several clinical benefits documented by observational and randomized controlled trials, including the control of blood pressure, extracellular volume, and phosphate levels and the reduced need for medication [1, 2]. Frequent HHD has been associated with a reduction in left ventricular hypertrophy and the stabilization of left ventricular ejection fraction [3, 4]. Observational studies have shown HHD to be associated with better survival than HD performed in centers and peritoneal dialysis (PD) [1, 5–7]. Other studies have shown that mortality rates are improving faster for HHD and PD than for hemodialysis (HD) performed in facilities [8, 9]. From a patient experience perspective, patients on HHD have reported higher quality of life and independence [10]. HHD is considered to be an economical way to manage end-stage kidney disease (ESKD) [11, 12].

The COVID-19 pandemic has had a significant impact on patients with ESKD and their care, especially given the potential for severe complications in those with depressed immune status. Patients receiving in-center HD have been particularly affected by the pandemic because of their need to travel multiple times a week to receive treatment. Patients on HHD may have an advantage over in-center HD patients because their risk of exposure to infection is lower at home than that of patients who spend time in a center for their dialysis treatments [13, 14].

At present, Chinese patients with chronic kidney disease account for 10.8% of the adult population, with up to 120 million patients, of whom approximately 2 million are patients with ESKD who need dialysis [15]. However, only 608,000 people are receiving dialysis treatment (including 553,000 receiving HD and 55,000 receiving PD), and the treatment rate for uremia is only 20-25% [16]. Moreover, in mainland China, all HD treatments are performed in centers, and the number of HHD patients is zero. HHD is a good solution.

Our hospital received the permission of the Shanghai government to carry out HHD in 2018. Then, we set up the first and only HHD center in mainland China. Our first HHD patient returned home to start independent HD on April 27, 2020. There were six HHD patients in our center; three are receiving HHD, and four are still undergoing treatment at the center. The purpose of this study was to explore the efficacy and safety of HHD in the treatment of Chinese patients with ESKD.

## Participants and methods

### Patients

We enrolled HD patients from the dialysis center in Renji Hospital, Shanghai, China. We have more than 200 incentre hemodialysis patients.

At first, we conducted a questionnaire on HHD in our center to investigate the intention of patients for this project. Among the patients who intended to participate in HHD, we chose the appropriate ones. The inclusion criteria were patients aged 18-75 years, with good fistula function (blood flow ≥200 ml/min), the propensity for self-management, and a living environment suitable for HHD. Informed consent was obtained from each patient.

## Methods

### Establishment of an HHD training center

The study was approved by the Ethics Committee of Renji Hospital under ethical approval number KY2020-006. All methods were carried out in accordance with the Declaration of Helsinki. The training center was approved by the Chinese government.

Experienced doctors, nurses and engineers were selected to establish the HHD team. The team members were sent to HHD training centers in Australia, New Zealand, the United States and Hong Kong, where HHD is advanced, to learn the HHD technology and training methods. Team members then formulated rules and regulations, management specifications and emergency plans according to their responsibilities, the safety and emergency measures system, troubleshooting for patients, follow-up and retraining.

### Vascular access

Vascular access with good function was established. Nurses guided patients to adopt buttonhole cannulation in our HHD center.

### Patient training

Doctors, nurses and engineers trained and assessed patients separately.

The training of doctors included the principles of HD, the role of heparin, the use of phosphorus binding agents and other drugs, diet control, blood pressure control and other HD-related knowledge.

Nurses formulated training plans for patients that included demonstrations and guidance on flushing the dialyzer and pipeline, fistula cannulation, monitoring vital signs and treating emergencies during loading, unloading and dialysis, disposing of medical wastes and troubleshooting. The patients were also trained in the theoretical knowledge of HD, including sterility, vascular access, daily diets and nutrition.

Engineers trained the patients on handling the HD machine alarm, using the water treatment equipment and monitoring dialysis water quality.

After the training, an assessment was conducted using written and oral forms.

### Patient monitoring

There are three forms of HHD visits: home visits, remote internet monitoring, and outpatient services.

Home visits were carried out by nurses and engineers at least once a week during the first month of treatment and at least once a month thereafter. The frequency of home visits was increased as needed. The contents of the visit included checking the patient’s daily dialysis records; inquiring about clinical symptoms, dialysis-related information (fistula cannulation, machine installation, dialysis prescription implementation and dialysis complications) and medication; and taking biological samples from patients when necessary. Water quality was tested at least once a month.

The dialysis process was monitored in real time via the internet and a 24-hour online telephone service. Professional staff and patients used visual software and high-definition cameras to conduct audio–video conference calls. At the same time, during the dialysis process and interval, the patients could also communicate with the medical staff not only by using a telephone or smart phone but also by WIFI so that real-time communication could be established with the smartphone application. Therefore, treatment options could be suggested and provided immediately. Medical staff could inform patients or relevant personnel in a timely manner and provide corresponding medical services based on an analysis of the patient’s background when adjustment was needed.

The patients performed the treatments in working hours. If they want to perform the treatments in non-working hours, they can call the center at first and we can monitor them online.

Patients also used hemodialysis machines with automatic data collection and a transmission function module for dialysis. The Internet was used to collect and record data as follows: (1) Before treatment, the hemodialysis treatment prescription, including treatment start time and treatment parameter settings (blood flow, dialysate flow, dehydration preset value, treatment time preset value). (2) During the treatment, arterial pressure, venous pressure, transmembrane pressure, blood pressure, and heart rate were recorded every hour. (3) At the end of treatment, actual dehydration, blood pressure was recorded.

Outpatient visits to the hospital were made at least once a month.

### Water quality requirements

Dialysis water treatment equipment shall have registration certificate and production license. The quality of water produced by dialysis water treatment equipment shall meet the requirements of YY0572-2015, which is the same as hemodialysis center.

The contents of micro-bacteria and endotoxin were measured every month, chlorine was measured every half year, and heavy metals were measured every year by engineers and nurses. The patients performed chemical tests for residual chlorine and the hardness of the dialysis water from the water treatment system before every dialysis by themselves.

### Clinical data and laboratory measurements

Demographic and medical data included age, sex, blood pressure, and dialysis-related data. Laboratory tests were conducted in our central testing laboratory, including hemoglobin (Hgb), serum creatinine (Cr), urea nitrogen (BUN), uric acid (UA), albumin (Alb), calcium (Ca), phosphorus (P), parathyroid hormone (PTH), and brain natriuretic peptide (BNP) levels.

### Hemodialysis equipment

We used a Fresenius 5008 s for HD and a Fresenius AquaWTU125 for water treatment. Fresenius (FX80) hollow-fiber HD filters were used in HD, and Fresenius (FX1000) hollow-fiber HD filters were used in hemodiafiltration (HDF).

### Statistical method

SPSS 21.0 software was used for statistical analysis. Population data are expressed in constituent ratios. Continuous variables with a skewed distribution are expressed as the median (interquartile range).

## Results

### In the training center

Six patients who underwent regular dialysis in the HD center of our hospital were selected for HHD training. The clinical data of the patients are shown in Table 1, including 4 males and 2 females. The mean age of the patients was 47.5 (34.7-55.7) years, and the mean dialysis age was 33.5 (11.2-41.5) months. Dialysis was conducted by HDF for 4 h three times per week using autologous arteriovenous fistula, low molecular weight heparin anticoagulation, and total ultrafiltration not exceeding 600 ml per hour.

**Table 1:** Basic information of the home hemodialysis patients

	Patient 1	Patient 2	Patient 3	Patient 4	Patient 5	Patient 6
Sex	male	female	male	male	female	male
Age	58	44	55	35	34	51
Primary disease	Hypertensive nephrosclerosis	nephritis	Hypertensive nephrosclerosis	nephritis	nephritis	nephritis
Dialysis age (months)	58	32	35	36	13	6
Training time (months)	15	17	24	30	13	6
Time to go home (months)	24	12	6	/	/	/

### HHD treatment

After training, 3 patients returned home to begin independent HD. The HHD dialysis prescription was 4 h of HD three times per week in the first 2 months. After 2 months, HDF was performed 3 times per week. In 3 patients who did not return home, one of them will return home 1 month later, the other two are still training in center.

Autologous arteriovenous fistula was used, low molecular weight heparin anticoagulation was administered, and the total ultrafiltration volume was no more than 600 ml per hour. Patients performed dialysis machine cleaning, assembly and testing, dialysis fluid preparation and examination, fistula cannulation and vital sign monitoring independently. Before dialysis, simple chemical tests of residual chlorine and hardness were carried out on the water used in the water treatment system. The water processor and HD machine were cleaned every day. The entire procedure was supervised by professionals, and the patient was always able to consult a dedicated engineer. Professional doctors, nurses, and engineers made home visits on the first day of HHD, once a week in the first month, and once a month beginning in the second month. The comparison of biochemical results before and after HHD treatment is shown in Table 2. The laboratory indicators of all patients, including Hgb, Alb, Ca, P, PTH, BNP, and β2-MG levels, remained stable before and after HHD treatment.

**Table 2:** Biochemical indices of home hemodialysis patients before and after returning home

	Before HHD training						Before returning home			6 months later			12 months later			24 months later		
	Patient 1	Patient 2	Patient 3	Patient 4	Patient 5	Patient 6	Patient 1	Patient 2	Patient 3	Patient 1	Patient 2	Patient 3	Patient 1	Patient 2	Patient 3	Patient 1	Patient 2	Patient 3
**Blood flow (ml/min)**	**260**	**210**	**250**	**250**	**260**	**250**	**260**	**210**	**250**	**260**	**210**	**250**	**260**	**210**	**/**	**260**	**/**	**/**
**Dialysate flow (ml/min)**	**500**	**500**	**500**	**500**	**500**	**500**	**500**	**500**	**500**	**500**	**500**	**500**	**500**	**500**	**500**	**500**	**/**	**/**
**Kt/V**	**1.4**	**1.3**	**1.3**	**1.3**	**1.2**	**1.3**	**1.44**	**1.57**	**1.7**	**1.40**	**1.42**	**1.6**	**1.42**	**1.42**	**/**	**1.53**	**/**	**/**
**URR**	**70**	**63**	**56**	**60**	**65**	**70**	**71**	**75**	**75**	**70**	**72**	**75**	**70**	**72**	**/**	**72**	**/**	**/**
**Hemoglobin (g/L)**	**111**	**123**	**130**	**116**	**120**	**115**	**117**	**100**	**108**	**138**	**108**	**106**	**114**	**108**	**/**	**131**	**/**	**/**
**Erythropoietin dose (weeks)**	**10,000 u**	**10,000 u**	**10,000 u**	**10,000 u**	**10,000 u**	**10,000 u**	**10,000 u**	**10,000 u**	**10,000 u**	**10,000 u**	**10,000 u**	**10,000 u**	**10,000 u**	**10,000 u**	**/**	**7000 u**	**/**	**/**
**Albumin (g/L)**	**40**	**37**	**40.7**	**39**	**40**	**39**	**41.8**	**40.1**	**36.4**	**45.4**	**41.4**	**39.6**	**44.5**	**41.4**	**/**	**43.8**	**/**	**/**
**Calcium (mmol/L)/phosphorus (mmol/L)**	**2.4/1.57**	**2.4/2.55**	**2.3/1.54**	**2.4/1.67**	**2.3/1.8**	**2.5/1.9**	**2.4/1.21**	**2.42/2.05**	**2.34/1.41**	**2.30/2.46**	**2.33/2.05**	**2.35/1.69**	**2.46/1.87**	**2.33/2.05**	**/**	**2.46/1.89**	**/**	**/**
**Parathyroid hormone (pg/ml)**	**38**	**281**	**123**	**300**	**150**	**200**	**94.4**	**181**	**123**	**247**	**177**	**102**	**152**	**177**	**/**	**78.1**	**/**	**/**
**Types of antihypertensive drugs**	**4**	**1**	**3**	**1**	**2**	**2**	**4**	**1**	**3**	**4**	**1**	**3**	**4**	**1**	**/**	**4**	**/**	**/**
**Residual renal volume (ml/day)**	**500**	**1000**	**500**	**800**	**500**	**1000**	**500**	**1000**	**500**	**500**	**1500**	**500**	**500**	**1500**	**/**	**300**	**/**	**/**
**Brain natriuretic peptide (pg/ml)**	**350**	**71**	**227**	**250**	**45**	**150**	**135**	**95**	**56**	**458**	**127**	**95**	**71**	**127**	**/**	**82**	**/**	**/**
**Beta 2 microglobulin (Before/After hemodialysis)**	**33/5.6**	**27/9.7**	**23/4.5**	**23/9.5**	**28/4.5**	**25/5.1**	**33.1/5.5**	**26.9/9.9**	**22/4.2**	**34.2/16.3**	**28.6/10.3**	**21.8/4.8**	**36.1/13.3**	**28.6/10.3**	**/**	**38.9/7.2**	**/**	**/**

*Kt/V* urea clearance index, *URR* urea decline rate, EPO: erythropoietin

### Troubleshooting

During the follow-up, there were no serious adverse events leading to hospitalization or death, but there were several adverse events (Table 3).

**Table 3:** Troubleshooting

	Immediate Cause of Adverse Event	Time (days)	Details	Processing method
**Patient 1**	Fistula puncture	26	The intima of the blood vessels thickened	1. Reconstruction of the arterial tunnel 2. Remote instruction for injection
	The chlorine test of reverse osmosis water was unqualified	1	The chlorine in the tap water had increased	Installed carbon canister
	Bicarbonate supply line air alarm	8	Winter indoor temperature was low, B powder in B-bag was hard to dissolve	Changed to B liquid
	Fistula puncture	8	Arterial puncture site flap formation	Adjusted puncture direction
	Water treatment leakage	1	One of the valves was not tightened	Engineer arrived to handle the problem
**Patient 2**	Fistula puncture	1	Psychological factors	Adjusted puncture direction
	Transient hypotension and syncope	1	The ultrafiltration setting was incorrect	Video guidance, blood return with the help of family members
	The hardness of dialysis water exceeded the standard	6	Tap water quality was hard	Added a softening device
	Water treatment leakage	1	The booster pump overheated	Changed the booster pump
	Arterial pressure alarm during hemodialysis and difficult injection.	30	Hyperplasia of the subcutaneous tunnel intima	Returned to the dialysis center, Reconstructed the tunnel
**Patient 3**	Coagulation	1	Forgot the heparin	Finished the dialysis manually under the guidance of a nurse by internet remote monitoring

Among these adverse events, fistula injection problems were the most common and occurred four times in two patients. The nurses solved the problems online by monitoring the process remotely, making extra home visits or requiring the patient to return to the dialysis center.

Two of the adverse events were caused by human error. In case 2, the patient showed symptoms of hypotension during remote monitoring. The nurse worked with family members by phone to give additional guidance about blood return. The doctors found that the ultrafiltration setting was incorrect. After a meeting with staff, preventive measures were established. Before each HHD session, the patients were required to take pictures of the HD machine settings to share with the responsible nurse who then confirmed the settings. The other human error was coagulation of the line and filter when the patient forgot to add heparin. The patient finished the dialysis manually under the guidance of a remote nurse. After a meeting, the staff decided the way to avoid this problem was to have the patient double check the medication before each therapy session.

In addition to fistula problems and human error, there were machine defects, including dialysis water, bicarbonate supply line air alarms and water treatment leakage. The problems were resolved quickly by technicians through extra home visits.

## Conclusion

Combined with professional training by doctors, nurses, engineers and various forms of visits, including home visits, remote internet monitoring, and outpatient services, HHD is feasible and safe for ESKD in mainland China. However, larger-scale and longer-term studies are needed to further confirm these findings.

## Discussion

China has a vast territory and a large population, but HD facilities and quality medical resources are limited. HHD is an ideal solution. Some researchers believe that HHD is the best kidney replacement therapy other than kidney transplantation. A current observational study showed that the mortality rate of HHD continued to be lower than all other modalities, including facility HD, automated peritoneal dialysis (APD), and continuous ambulatory peritoneal dialysis (CAPD), and was reasonably stable [11].

At the end of 2019, a novel coronavirus was identified as the cause of a cluster of pneumonia cases in China. Subsequently, the infection spread throughout the world, resulting in a global pandemic. Patients with ESKD are particularly vulnerable to severe COVID-19 due to older age and related comorbidities, such as diabetes and hypertension, in this population [17–22]. Of the patients with ESKD, patients who receive dialysis at home may be at a lower risk of developing COVID-19 than patients receiving in-center HD [23–26]. This was demonstrated in one study in which patients receiving in-center dialysis had an approximately twofold greater risk of infection than patients receiving home dialysis [24]. HHD is of practical significance in ensuring medical safety and reducing cross-infection and the consumption of medical resources as the COVID-19 pandemic becomes normalized.

HHD is generally considered very safe, with low adverse event rates similar to home HD and conventional in-center HD [27, 28]. During our follow-up, there were no serious adverse events leading to hospitalization or death. All of the problems were found through remote monitoring and were resolved quickly online or through extra home visits by staff. Our study suggests that successful HHD programs require doctors, nurses and engineers with HHD training qualifications. We sent medical staff to Australia, New Zealand, the United States and other countries with advanced HHD technology to learn about HHD technologies. Patient selection is also important. Relevant factors for patient selection include their living environment and facilities, their learning ability and motivation, the anxiety levels of the patients and their family members, and the type and severity of physical illness. Motivation and compliance are the most important factors. In addition, adequate preparation is particularly important to ensure the safety of HHD projects. We formulated rules and regulations, management specifications and emergency plans according to the responsibilities of team members, safety and emergency measures systems, troubleshooting for patients, follow up and retraining. Finally, with a 24-hour online telephone service, the dialysis process was remotely monitored in real time. Remote monitoring systems can help staff recognize potential problems that patients may be encountering at home before they develop into more serious issues; staff can also be readily available to troubleshoot acute complications with access or mechanical complications via a video connection [29]. In addition, staff need to make regular home visits to find and solve problems immediately.

Among the troubleshooting procedures, fistula injection problems were the most common and occurred four times in three patients. The nurses solved the problems online by remote monitoring and made extra home visits or required the patients to return to the dialysis center. We worked in a multidisciplinary way. Vascular surgeons examined the fistulas regularly and performed fistula ultrasounds every 3 months. All of our patients underwent buttonhole cannulation. Painful needling, fear of needles and difficulty cannulating are the most common challenges with needling in HHD [30]. We chose buttonhole cannulation because we thought it might allow for a shorter training time and be easier for the patients. Moreover, fistulas with short workable segments, small diameters, or deep positions are difficult to cannulate consistently without the buttonhole technique [30, 31]. However, buttonhole cannulation is associated with an increased risk of infection, so nurses must train patients in aseptic procedures.

Another major trouble in HHD patients is hypotension. There are several strategies for us to monitor and preventing it. In our center, ultrafiltration of HHD patient is suggested to be less than 600 ml per hour. If the ultrafiltration target exceeds the upper limit, the patients need to extend the dialysis time or add a hemodialysis the next day. We gave education to patients and ask them to control the weight gain during dialysis. During hemodialysis, blood pressure is detected by camera and hemodialysis machine. If the blood pressure is lower than the normal level, doctors will discuss with the patient how to adjust the medication for hypertension or dry weight in time. We also detected the blood BNP and pro-BNP of patients regularly to adjust dry weight.

Typically, the training for patients are 8-10 weeks at HHD program in other countries. There are several reasons for our patients requiring more time of training. First, this is the first time of HHD in mainland China. Doctors, nurses and engineers trained and assessed patients separately. The standard for patients to return home is very strict and conservative due to safety. Secondly, because of COVID-19, HHD training was interrupted and some communities were locked down and the patients’ return home were delayed. Finally, it took time to apply for and purchase hemodialysis equipment.

As COVID-19 becomes normalized, uremic patients will remain more susceptible to the virus than healthy people. HHD is of practical significance in ensuring medical safety and reducing cross-infection and the consumption of medical resources. However, at present, in mainland China, all dialysis treatments are in-center, and the number of HHD patients is zero. This is the first attempt at home hemodialysis in mainland China. Our preliminary studies show that HHD is safe, effective and feasible. It is important to further strengthen quality management by establishing operating standards, which are necessary to reduce medical risks and maximize medical safety.

## Data Availability

The datasets generated and analyzed during the current study are not publicly available due to limitations of ethical approval involving the patient data and anonymity but are available from the corresponding author on reasonable request.
